# Bulk vs. Surface Structure of 3*d* Metal Impurities in Topological Insulator Bi_2_Te_3_

**DOI:** 10.1038/s41598-017-06069-3

**Published:** 2017-07-18

**Authors:** B. Leedahl, D. W. Boukhvalov, E. Z. Kurmaev, A. Kukharenko, I. S. Zhidkov, N. V. Gavrilov, S. O. Cholakh, P. Huu Le, C. Wei Luo, A. Moewes

**Affiliations:** 1 Department of Physics and Engineering Physics, University of Saskatchewan, 116 Science Place, Saskatoon, Saskatchewan S7N 5E2 Canada; 2 Department of Chemistry, Hanyang University, 222 Wangsimni-Ro, Seoul, 04763 Republic of Korea; 3 Theoretical Physics and Applied Mathematics Department, Ural Federal University, Mira Street 19, 620002 Yekaterinburg, Russia; 4 M.N.Mikheev Institute of Metal Physics of Ural Branch of Russian Academy of Sciences, S. Kovalevskoi 18 str., 620990 Yekaterinburg, Russia; 5 Institute of Physics and Technology, Ural Federal University, Mira 19 St., 620002 Yekaterinburg, Russia; 6 Institute of Electrophysics, Russian Academy of Sciences-Ural Division, 620016 Yekaterinburg, Russia; 7 Department of Electrophysics, National Chiao Tung University, Hsinchu, 30010 Taiwan, ROC; 8 Faculty of Basic Sciences, Can Tho University of Medicine and Pharmacy, 179 Nguyen Van Cu Street, Can Tho, Vietnam

## Abstract

Topological insulators have become one of the most prominent research topics in materials science in recent years. Specifically, Bi_2_Te_3_ is one of the most promising for technological applications due to its conductive surface states and insulating bulk properties. Herein, we contrast the bulk and surface structural environments of dopant ions Cr, Mn, Fe, Co, Ni, and Cu in Bi_2_Te_3_ thin films in order to further elucidate this compound. Our measurements show the preferred oxidation state and surrounding crystal environment of each 3*d*-metal atomic species, and how they are incorporated into Bi_2_Te_3_. We show that in each case there is a unique interplay between structural environments, and that it is highly dependant on the dopant atom. Mn impurities in Bi_2_Te_3_ purely substitute into Bi sites in a 2+ oxidation state. Cr atoms seem only to reside on the surface and are effectively not able to be absorbed into the bulk. Whereas for Co and Ni, an array of substitutional, interstitial, and metallic configurations occur. Considering the relatively heavy Cu atoms, metallic clusters are highly favourable. The situation with Fe is even more complex, displaying a mix of oxidation states that differ greatly between the surface and bulk environments.

## Introduction

The Bi-chalcogenides such as Bi_2_Te_3_ and Bi_2_Se_3_ have long been known for their thermoelectric properties^1, 2^, but recently they have gained a great deal of attention as three-dimensional topological insulators with a large band gap and a single Dirac cone on the surface^3–8^. Additionally, the introduction of magnetic impurities into Bi-chalcogenides can break time-reversal symmetry and open an energy gap at the Dirac point of the surface states^9–11^. Recently, Bi_2_Te_3_ and related systems with dilute doping of 3*d*-metal atoms (Ti, V, Cr, Mn, and Fe) have been found to have ferromagnetic transitions at low temperatures. Bi_2−*x*_Mn_*x*_Te_3_ was found to have a Curie temperature *T*_*C*_ around 10 K for x = 0.02^12^. For larger doping of thin films of Sb_2−*x*_V_*x*_Te_3_, with x = 0.35, *T*_*C*_ increases to 177 K and for SbCr_*x*_Te_3_ with x = 0.59 *T*_*C*_ increases to 189 K^13^ which is close to that of more traditional dilute magnetic semiconductors (DMS)^14^.

Most DMS materials are crystallized in zinc-blende or wurtzite crystal structures with tetrahedral bonds between atoms. In this respect Bi_2_Te_3_ is different, its tetradymite-type structure has atoms that form in octahedral coordination. When transition metal atoms are introduced to the tetradymite-type structure, there are various possible oxidation states they may exist in. In addition, the spin-orbit interaction is more complicated due to the expanded octet bonding where not only *p*-orbitals, but also *d*-orbitals join the hybridization. The relatively sophisticated tetradymite structure leaves a lot of structural and chemical factors available to be tweaked. This induces even more complicated structural configurations when impurity atoms are introduced, and as a result, create new, unexpected electronic and magnetic properties. In the present paper the local crystal and electronic structure of 3*d*-impurities (Cr, Mn, Fe, Co, Ni, Cu) in Bi_2_Te_3_ thin films are studied using x-ray photoelectron spectroscopy (XPS), x-ray ray absorption (XAS), and resonant x-ray emission (RXES) techniques. The experimental spectra are compared with density functional theory (DFT) calculations of formation energies for different configurations of the dopant atoms. These techniques are powerful in that they are non-invasive and able to distinguish between bulk and surface states, key to the study of topological insulators^15^.

## Experiment and Calculation Details

**Figure 1:**
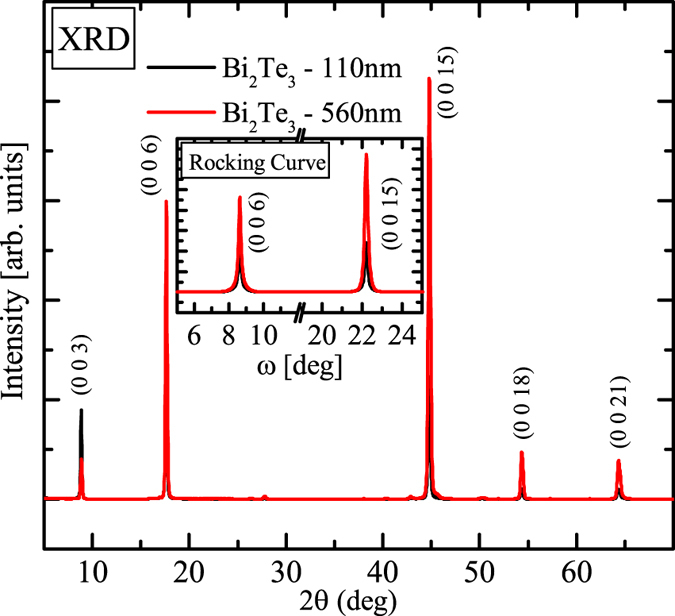
X-ray diffraction results show the high quality crystallinity of the Bi_2_Te_3_ thin films created using pulsed laser deposition.

Bi_2_Te_3_ thin films were grown on an Al_2_O_3_(0001) substrates via pulsed laser deposition using a stoichiometric Bi_2_Te_3_ target with a purity of 99.99%^16^. The KrF-pulsed laser fluence and repetition rate were 3.7 J/cm^2^ and 4 Hz, respectively. The base pressure of the growth chamber was below 2 × 10^−6^ Torr. Before loading into the chamber, the substrates were first cleaned using ultrasonication in acetone, methanol, and then deionized water for 30 minutes. The pressure in the chamber was maintained at 0.35 Torr while argon gas was introduced throughout the deposition process. The optimized substrate temperature was 260 °C. The deposition times were 5 minutes and 25 minutes to grow the films with thicknesses of approximately 110 and 560 nm, respectively. The orientation and crystallinity of Bi_2_Te_3_ films were determined using x-ray diffraction (XRD) with Cu *Kα* radiation (8048 eV); the results are displayed in Fig. 1.

The implantation of ions in Bi_2_Te_3_ thin film samples was carried out in a vacuum chamber that was evacuated to a residual pressure of 3 × 10^−3^ Pa. An ion beam with an energy of 30 keV was created at a source cathode by vaporizing the metal with an electric arc. The ions were then used to irradiate the sample in a pulsed mode (25 Hz). After 38 minutes of exposure the sample had an ion fluence (integrated flux over time) of 1 × 10^17^ cm^−2^. For ion implantation Cr, Mn, Fe, Ni, Co and Cu metals were used.

X-ray photoelectron spectroscopy (XPS) core-level measurements were performed using a spectrometer with an energy analyzer working in the range of binding energies from 0 to 1400 eV. The samples were introduced to vacuum (10^−7^ Pa) for 24 hours prior to measurement, and only samples whose surfaces were free from micro-impurities were measured and reported herein. The XPS spectra were recorded using Al K *α* (1486.6 eV) x-ray emission photons; the spot size was 100 *μ*m; the energy resolution is Δ*E* < 0.5 eV; and typical signal to noise ratios were greater than 10000:3. The x-ray absorption spectroscopy (XAS) measurements were taken at Beamline 8.0.1 at the Advanced Light Source (ALS), and the REIXS and SGM beamlines, the latter two being at the Canadian Light Source. Finally, the x-ray emission (XES) measurements were performed using Beamline 8.0.1^17^.

**Figure 2:**
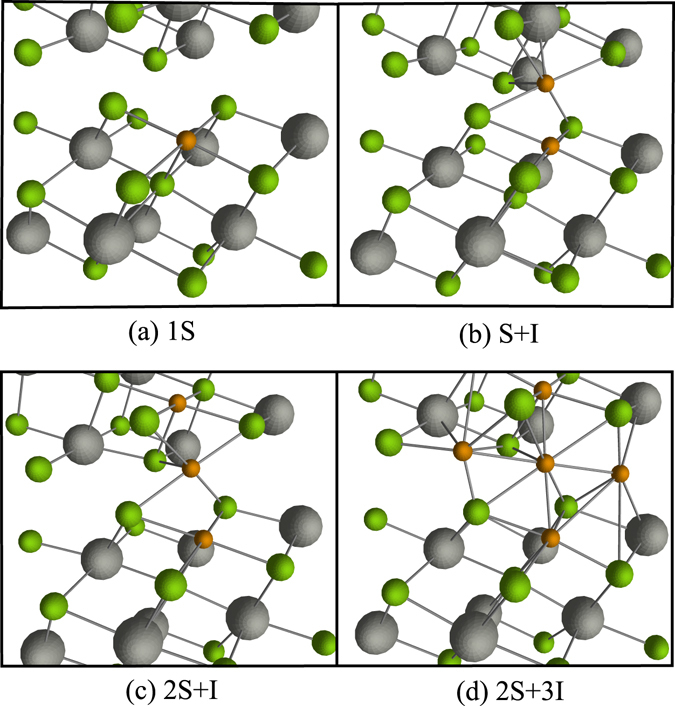
The four defect structures for which formation energies were calculated for in the Bi_2_Te_3_ crystal lattice. (**a**) 1*S* − a substitutional transition metal dopant residing in a Bi crystal site. (**b**) *S* + *I* − substitutial and interstitial atoms location adjacent to one another. (**c**) 2*S* + *I* − further clustering of metallic atoms. (**d**) 2*S* + 3*I* − this defect corresponds to large amount of metallic atoms clustering together, observed as the formation of metallic bonds. Bismuth atoms are displayed in grey, tellurium in green, and transition metal dopants in orange.

To simulate 3*d*-metal impurities in Bi_2_Te_3_ we applied the pseudo-potential code SIESTA^18^. For our purposes a supercell containing 60 atoms (Bi_24_Te_36_) was adequate; this provides a sufficient distance between embedded impurities. We calculated the formation energies for four possible combinations of impurities: a single substitutional impurity of bismuth (1*S*, Fig. 2a), a combination of substitutional impurity and embedded one into the interlayer space (*S* + *I*, Fig. 2b), a combination of two substitutional and one interstitial impurities (2*S* + *I*, Fig. 2c) and a configuration obtained by adding two interstitial atoms to the previous configuration (2*S* + 3*I*, Fig. 2d).

The crystal field multiplet calculations in this work use the algorithm initially formulated by Cowan, and the working code subsequently expanded on by Haverkort and Green *et al*.^19–22^. The free parameters include the crystal field strength (from which the local symmetry can be deduced), oxidation state, and the scaling of the intra-atomic Coulomb and exchange (Slater) integrals. The dipole transition matrix elements calculated by this code are then used in the Kramers-Heisenberg equation to simulate spectra^23^. All spectra are broadened by convolutions of a Lorentzian function (to simulate lifetime broadening), and a Gaussian function (to simulate experimental broadening) to match experimental conditions.

## XPS Measurements

Using Al K *α* photons, XPS allows one to probe the binding energies of a transition metal’s 2*p* electrons; this technique is primarily sensitive to the *surface* oxidation state of the atoms. The surface sensitivity is in the range of ≈5 nm, due to the mean free path of escaping electrons. Furthermore, if the metal atoms exist in multiple oxidation states simultaneously, we will observe a superposition of the peaks from the corresponding oxidation states in our sample. Of note is that Cr XPS data was not possible due to the overlap of Cr 2*p* and Te 3*d* binding energies, coupled with the dilute nature of Cr atoms, it was not feasible to subtract out the much stronger Te 3*d* signal.

**Figure 3:**
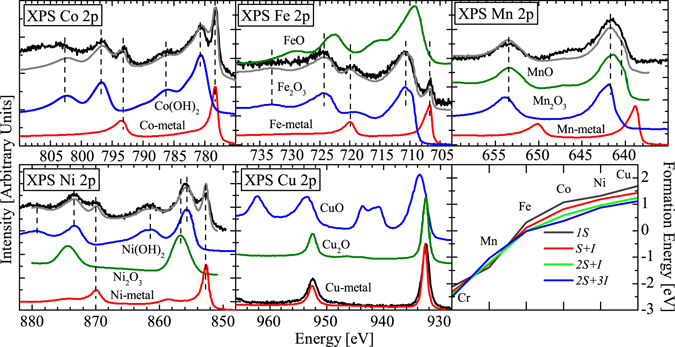
XPS spectra for doped samples. Black curves are 2*p* excitations of the TM dopant atoms in Bi_2_Te_3_. Grey curves are a linear combination of standard references. Table 1 indicates the approximate proportion of each oxidation state the dopants are in. The lower right panel are DFT calculated formation energies for each dopant for four types of defects.

Shown in Fig. 3 are our XPS measurements determining the 2*p*_3/2,1/2_ binding energies of the dopant atoms in Bi_2_Te_3_. For each dopant atom *except* Mn, there is an obvious contribution from the metallic phase of each element. This corresponds to the peak position in the metallic reference spectra also appearing in the doped Bi_2_Te_3_ samples (indicated with vertical dashed lines in each plot of Fig. 3). This agrees with what we would roughly expect to see when considering our calculations of formations energies (Table 1). For each element we see that the clustering of dopant atoms (and hence the formation of metal-like regions, corresponding to 2*S* + 3*I* and 2*S* + *I* defects), are quite favourable (see lower right panel of Fig. 3 for formation energies of defects), especially in the cases of the heavier elements Co, Ni, and Cu. These defects can be thought of as the tendency for the transition metal atoms to cluster together (see Fig. 2c,d). On the other hand, each doped sample also contains ionized dopants that reside in the Bi_2_Te_3_ lattice that do not form metallic clusters.

**Table 1:** The XPS spectra in Fig. 3 were decomposed into their component oxidation states through a linear combination of common oxides and metals.

	0 (metallic)	2+	3+
Cr	—	—	—
Mn	0	88	12
Fe	25	0	75
Co	46	54	0
Ni	30	68	2
Cu	100	0	0

The tabulated values in this table indicate the percentage of each oxidation state found for each dopant metal on the Bi_2_Te_3_ surface. The corresponding linear sum is also plotted in Fig. 3 as grey curves.

By normalizing the reference XPS spectra to have equal 2*p*_3/2_ and 2*p*_1/2_ edge jumps, we were able to take a linear combination of these to obtain good approximations for proportion of each oxidation state for each metal in Bi_2_Te_3_. The grey curves in Fig. 3 are the linear combination results; in all cases the match with the experimental spectrum is remarkably good. We have summarized the results and percentages of each oxidation state for each dopant in Table 1.

Manganese is specifically of interest because of its unique incorporation into Bi_2_Te_3_. When component oxidation states were analyzed, we found that there is virtually no metal-like signal, and 91% Mn^2+^. This means that in the case of Mn doping, the cation substitution Mn^2+^ → Bi^3+^) is the primary defect. Our DFT calculations in the lower right panel of Fig. 3 support this view for Mn as well. It is the only dopant for which the formation energy of a substitutional impurity is the most favourable. In general our DFT calculations support the observed XPS results for all dopants, with the difference in 1*S* and 2*S* + 3*I* formation energies corresponding to the likelihood of metallic cluster formation. That is, metallic clusters tend to form when the cluster-like 2*S* + 3*I* defects are sufficiently lower in energy than 1*S* defects, and the reverse is true as well, as shown in the case of manganese.

## X-ray Absorption and Emission

**Figure 4:**
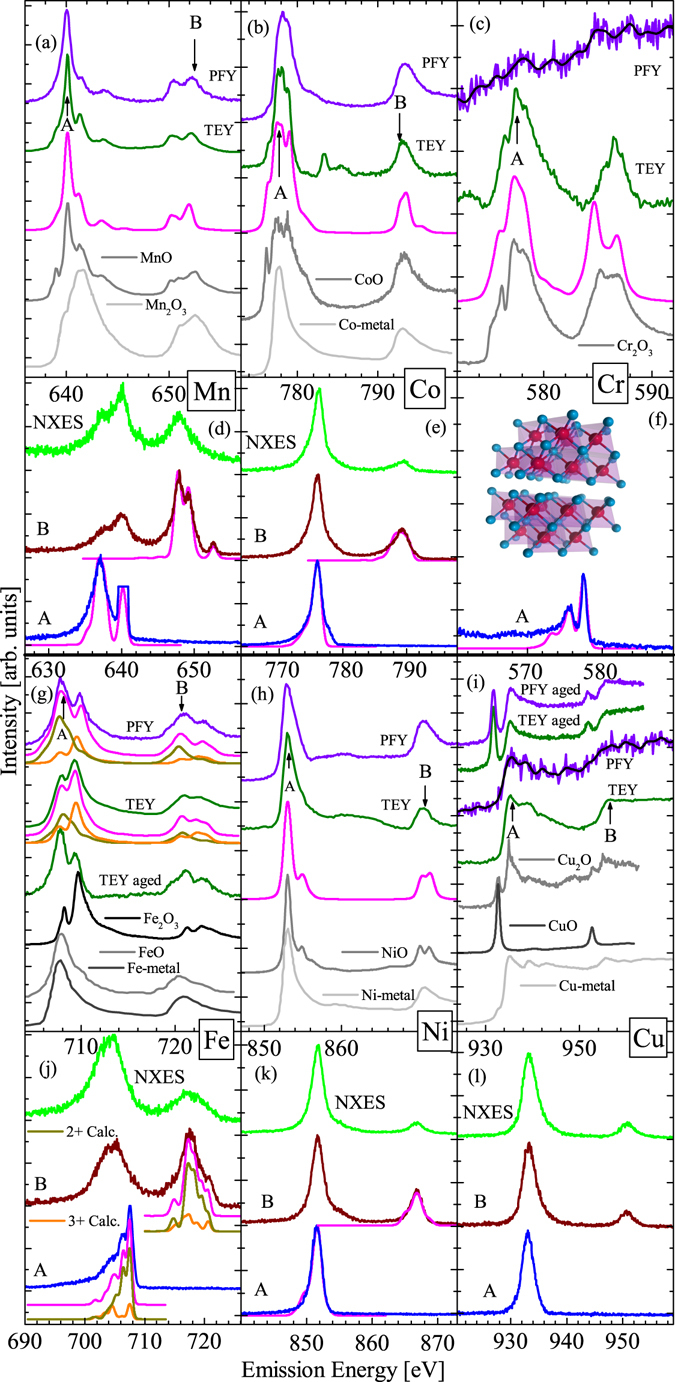
X-ray absorption and emission at the transition metal *L*-edges along with calculated spectra (pink). The Bi_2_Te_3_: TM XAS spectra are shown in black with standard oxide references in grey for comparison. Panels (a–c) and (g–i) contain the XAS spectra, while beneath each is the corresponding element’s XES spectra in panels d–f and j–l, respectively. In panel (g), the iron calculations are shown in orange and dark yellow, with the linear sum of the two shown in pink. A thorough discussion of each element’s spectra is given separately in the main text. High quality Ni, Co, Fe metal XAS reference spectra were taken from ref. 24.

Further x-ray measurements complement our XPS findings, these are displayed in Fig. 4. When coupled with crystal field calculations, these measurements can be used to deduce the local symmetry of the dopant ions, as well as their oxidation state. By achieving agreement between calculation and experiment, we can use the parameters used in the calculation to extract real physical results. Another important distinction to consider is that the XAS measurements were performed in both total electron yield (TEY) mode, only sensitive to the first ≈5 nm of the sample surface, and the more bulk sensitive partial fluorescence yield (PFY). XES measurements are inherently bulk sensitive (≈100 nm) similar to PFY, due to the greater escape depth of photons as compared to electrons.

For comparison, we have displayed the absorption spectra of common oxides and metals next to the spectra of each of the dopants in Fig. 4. Since each oxidation state for each 3*d* transition metal atom has a vastly different shape, this comparison alone can determine the ion’s valency. In each case the calculation (pink) was performed for the primary oxidation state that was found via the XPS data. In general, they adequately agree with the experimental spectra, despite the fact of the existence of some portion of pure metallic bonding in each sample. This is because metallic absorption spectra are rather broad and featureless, whereas the rich spectra of charged transition metal atoms still shines through on top of this. Below we will discuss each dopant in turn and some slight exceptions to the above general statements.

### Cobalt

In the case of cobalt (Fig. 4b), which has a high percentage of metallic bonding, the agreement between calculation and experiment is least satisfying (due to a large Co-metal contribution), Despite this, sharp scattering features can still be seen on top of the metallic signal in both TEY and PFY modes. In fact, the differences between these two spectra illustrate that cobalt in the bulk of the sample tends more towards a metallic state, while on the surface it is still largely found in its 2+ state. This is evident from the much more rich TEY spectrum, and in accordance with our XPS results above, which stated that 54% of Co atoms are in a 2+ state. From the agreement with our calculation we can deduce that cobalt in the the sample that is not metallic is in very nearly a perfect octahedral coordination. The splitting of the *e*_*g*_ and *t*_2*g*_ 3*d* orbital is given by the 10*Dq* parameter, and since 10*Dq* = 0.9 eV; *Ds* = *Dt* = 0, we can be sure it is octahedrally coordinated.

### Chromium

A similar analysis between the topological surface and deeper bulk environments for other dopants can also be done. In the case of chromium, the dopant atoms effectively *only* reside on the surface and are not incorporated into the host lattice at all during the ion implantation process, despite being subject to the same fluence. We have two pieces of evidence to support this: (1) the lack of any meaningful PFY signal; in Fig. 4c the PFY is extremely weak; (2) the XES had an order of magnitude less counts per second for Cr as compared to the other samples (hence why only one excitation is shown in Fig. 4f; it was not possible to obtain more data due to the time length of the measurements). In both cases it is likely that the emitted photons almost entirely originate from the thin surface layer (in which Cr^3+^ is nicely substituted into Bi_2_Te_3_). Hence, the bulk of the sample must have been effectively void of Cr ions.

As evidenced by the strong TEY signal, we are certain that the surface contains Cr^3+^ ions in a slightly warped octahedral environment (see Table 2 for crystal field parameters). Therefore, we have found a profound difference in the assimilation environments for Cr atoms in Bi_2_Te_3_. Cr atoms appear completely comfortable in a 3+ octahedral surface environment, but will not form in the bulk of the crystal lattice. The lack of bulk Cr can possibly be explained by its small ionic radius, because it is smaller than the other transition metal dopants used here, it may have difficulty finding a low energy state to reside in, and it is effectively expelled from the lattice.

**Table 2:** Shown are the crystal field parameters for the calculations in pink in Fig. 4.

	10D*q*	*Ds*	*Dt*	*β*
Cr	1.65	−0.05	−0.05	0.75
Mn	0.6	0	0	0.72
Fe^2+^	1.0	0	0	0.7
Fe^3+^	1.6	−0.02	−0.02	0.6
Co	0.9	0	0	0.75
Ni	1.3	0	0	0.75
Cu	—	—	—	—

The units for 10*Dq*, *Ds*, and *Dt* are eV, while *β* is unitless and corresponds to the scaling of the interatomic Slater integrals.

### Nickel

Nickel doping is quite similar to cobalt in that a substantial fraction of the dopant atoms prefer to metallically cluster together. However, in this incidence the TEY and the PFY for Ni (Fig. 4h) show nearly identical results, with spectra that are a mix between that of Ni-metal and NiO, in agreement with the XPS data. That is, there is a substantial proportion of metallic Ni clusters present. The main conclusion here is that there is no difference between the surface and bulk Ni incorporation, in both locations effectively identical results were found.

### Manganese

The most consistent dopant is manganese. In this case all of the DFT, XPS, TEY, and PFY results support the same conclusion: that Mn^2+^ is integrated into the host crystal in substitutional octahedral positions, but in an environment with a much lower crystal field strength than MnO (which is 1.0 eV)^25^, due to the larger spatial environment the Mn ions have in our case. In a past study it was shown that pristine Bi_2_Te_3_ and Mn doped Bi_2_Te_3_ have have measurably different conductive properties^26^. This difference must not originate due to metallic Mn, and in accordance with the cited study, there is no need to invoke any special inherent surface states to explain the conductive behaviour in the doped sample. Effectively, when incorporating Mn into Bi_2_Te_3_, both the surface and the bulk environments react identically, and any topological differences must be a result of the host material itself.

### Copper

We found that copper undergoes a large scale transformation with age in Bi_2_Te_3_. Initially it was found to be in a purely metallic state; this was confirmed by the XPS measurements as well as both TEY and PFY in Fig. 4i. However, upon exposure to atmospheric conditions for one year, a substantial fraction of the metallic clusters have converted to 2+ ions. This is evident from the followup absorption spectra taken (shown as “aged”), which show substantial contributions to the signal in the same energetic locations as that of CuO. We believe we can dismiss simple oxidation and the formation of CuO because the bulk of the sample also shows this same phenomena, and the exposure with air to the bulk of the sample is negligible. Also, surface oxidation generally takes place on the scale of only a few minutes^27^, and the initial XPS and XAS measurements showed no sign of this (after the samples were already exposed to air for months). Hence, we can conclude that the bulk and surface properties of Cu dopants in Bi_2_Te_3_ are effectively identical, and furthermore they age and change identically, and that no special surface states for Cu ions exist upon ion implantation. Note that copper spectra show no fine multiplet structure because of its full 3*d* shell (the same holds for *d* ^9^ in the case of Cu^2+^), so calculations offer no extra insight in this specific case.

### Iron

Lastly, iron is the most complicated case as it was necessary to use a combination of oxidation states on both the surface and in the bulk. However, the ratio of 2+:3+ Fe ions on the surface and in the bulk of the material is much different. If we first consider the TEY in Fig. 4g and compare it to the XPS in Fig. 3 we discover that there are some conflicting results. To contrast these, we examine further XAS measurements done a year after the initial ones to reveal that the surface undergoes a slow aging process. Initially, our XPS results showed that the surface contained 75% Fe^3+^ and 25% metallic Fe (Table 1). Then upon aging for six months our TEY measurements indicate that this ratio has fallen to about 58% Fe^3+^, while the remaining 42% can be classified as Fe^2+^. This is apparent from our linear sum of Fe^2+^ and Fe^3+^ calculations in Fig. 4g; one can see that the sum of the calculations is a near perfect fit for the experimental TEY spectrum. To test for reproducibility, further TEY measurements were done another year later (TEY aged), and they unambiguously show that the iron on the surface of the sample has become nearly entirely 3+. Hence, we have shown that the Bi_2_Te_3_ surface enables a slow transformation of Fe atoms’ oxidation state toward 2+ , given sufficient time.

What is interesting is how the bulk of Bi_2_Te_3_ reacts in a manner very contrary to its surface. In this case, bulk sensitive PFY measurements taken 18 months apart show no change. We also found that contrary to the surface, it is Fe^2+^ ions that are much more inclined to reside in the bulk. To illustrate this, a similar analysis as above was done. The 2+ and 3+ calculations together show that the ratio of 2+:3+ is 60:40 in the bulk. It appears clear that in the case of iron dopants in Bi_2_Te_3_ the surface states are vastly different from that of those in the bulk, and undergo different incorporation mechanisms, as well as different aging effects.

## Conclusion

In closure, we have studied the differences between dopant atoms on the surface and in the bulk of what is known to be a promising material for topological technologies. The inherent differences between the bulk and surface structural properties can manifest themselves upon examining how transition metal dopants assimilate into the host lattice. This method of doping with 3*d* metals is an oft use way of fine-tuning a material’s electronic and magnetic properties, and certainly shows favourable evidence to do so here again. Each of our dopants Cr, Mn, Fe, Co, and Ni behaved differently in Bi_2_Te_3_, facilitating the idea that there is a great deal of freedom in fine-tuning this versatile material. Thus, Bi_2_Te_3_ may be further honed by exploiting this large degree of freedom via synthesis using other techniques and/or introducing the dopants during synthesis. This would undoubtedly open the door to many other possibilities exploiting this material. By retaining the traditional surface conductivity and bulk insulating properties of this topological insulator, while tweaking its electric and magnetic properties to be better suited for a given technology, one is able to hone this widely heralded material for a tremendous array of technologies.
